# Pitfall of isolated superior mesenteric artery dissection with normal D‐dimer level

**DOI:** 10.1002/ccr3.6060

**Published:** 2022-07-22

**Authors:** Sumika Uno, Shun Yamashita, Masaki Tago, Shu‐ichi Yamashita

**Affiliations:** ^1^ Faculty of Medicine Saga University Saga Japan; ^2^ Department of General Medicine Saga University Hospital Saga Japan

**Keywords:** contrast‐enhanced computed tomography, D‐dimer, isolated superior mesenteric artery dissection, sudden‐onset abdominal pain

## Abstract

A 51‐year‐old man with untreated hypertension developed sudden‐onset epigastric pain*.* Despite a normal D‐dimer level, abdominal contrast‐enhanced computed tomography revealed superior mesenteric artery dissection. Abdominal contrast‐enhanced computed tomography is mandatory when examining patients with sudden‐onset abdominal pain, even those with a normal D‐dimer level.

## Graphical abstract

The D‐dimer level can be normal in patients with isolated superior mesenteric artery dissection. Abdominal contrast‐enhanced computed tomography is essential for patients with sudden‐onset abdominal pain, even those with a normal D‐dimer level.

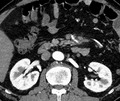

A 51‐year‐old man with untreated hypertension awoke with epigastric pain, which disappeared after a few minutes. Three days later, he was transferred by ambulance to our hospital because sudden severe epigastric pain appeared when picking up a baseball. On admission, his blood pressure was 256/147 mmHg. He had epigastric tenderness and exhibited profuse cold sweating. Despite his normal D‐dimer level of 0.46 μg/ml, abdominal contrast‐enhanced computed tomography (CT) revealed superior mesenteric artery (SMA) dissection (Figure 1). With maintenance of his systolic blood pressure below 120 mmHg, he was discharged on the ninth hospital day without an increase in the size of the false lumen on abdominal contrast‐enhanced CT.

**FIGURE 1:**
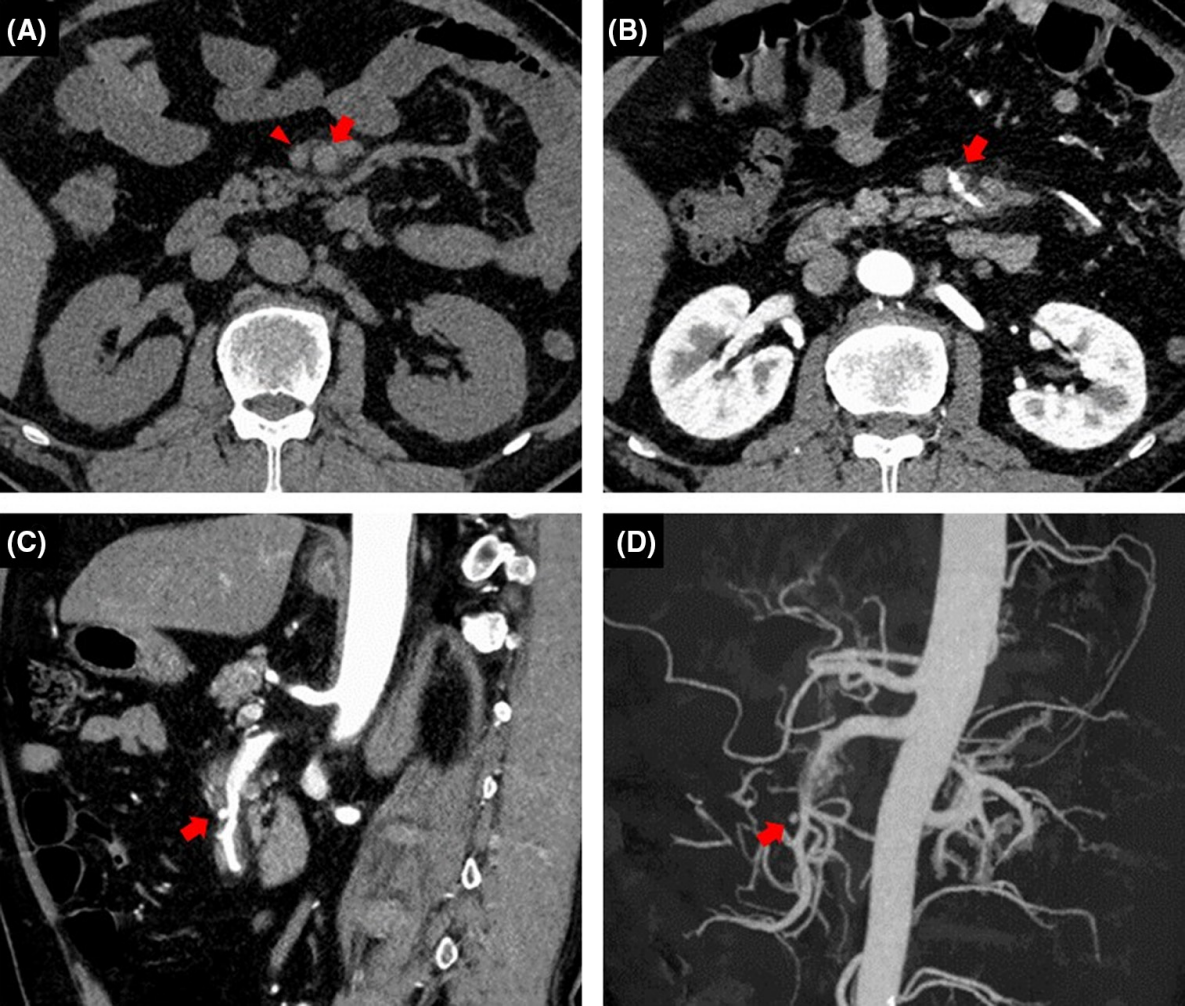
Findings of abdominal computed tomography (CT) and CT angiography on admission. (A) Axial imaging without contrast enhancement. (B) Axial imaging with contrast enhancement. (C) Sagittal imaging with contrast enhancement. (D) Three‐dimensional abdominal CT angiography. (A) Axial abdominal CT imaging without contrast enhancement showed the dilated superior mesenteric artery (SMA) (arrow) with the relatively decreased superior mesenteric vein (SMV) (arrowhead), resulting in the so‐called “smaller SMV sign.” (B–D) Horizontal and sagittal abdominal CT imaging with contrast enhancement and three‐dimensional CT angiography showed the formation of a false lumen in the SMA (arrow). These findings indicated the presence of dissection in the SMA.

The incidence of spontaneous isolated SMA dissection (ISMAD) is as low as 0.06%.^1^, ^2^ Although abdominal contrast‐enhanced CT is required to diagnose ISMAD, it is not always performed in patients with a normal D‐dimer level because of the extremely high negative predictive value of the D‐dimer level for acute aortic dissection.^1^ However, the D‐dimer level in patients with ISMAD can be normal.^1^ Because sudden‐onset abdominal pain appears in 92% of patients with ISMAD,^2^ abdominal contrast‐enhanced CT is mandatory even in patients with a normal D‐dimer level who exhibit such symptoms.

## AUTHOR CONTRIBUTIONS

SU involved in concept, literature search, and drafting of manuscript. SY involved in concept and drafting of manuscript. MT involved in concept and literature search. SI‐Y involved in concept and revision of manuscript.

## FUNDING INFORMATION

No specific grant was received for this work from any funding agency.

## CONFLICT OF INTEREST

The authors state that they have no conflict of interest.

## CONSENT

Written informed consent was obtained from the patient to publish this report in accordance with the journal's patient consent policy.

## Data Availability

The data that support the findings of this study are available from the corresponding author upon reasonable request.

## References

[1] Tanaka Y , Yoshimura T , Kimura K , et al. Clinical characteristics of spontaneous isolated visceral artery dissection. J Vasc Surg. 2018;67(4):1127‐1133. 29056349 10.1016/j.jvs.2017.08.054

[2] Kim YW . Current understandings of spontaneous isolated superior mesenteric artery dissection. Vasc Specialist Int. 2016;32(2):37‐43. 27386450 10.5758/vsi.2016.32.2.37 PMC4928602

